# A Histologically Complete Response to Immunotherapy Using Pembrolizumab in a Patient with Giant Cell Carcinoma of the Lung: An Additional Report and Literature Review

**DOI:** 10.1155/2019/1763625

**Published:** 2019-10-15

**Authors:** Tomoo Kakimoto, Mamoru Sasaki, Tatsuya Yamamoto, Arifumi Iwamaru, Kentaro Ogata, Ko Lee, Shingo Nakayama, Naoto Minematsu

**Affiliations:** ^1^Department of Internal Medicine Hino Municipal Hospital, Hino-shi, Tokyo, Japan; ^2^Department of Surgery, Federation of National Public Service Personnel Mutual Aid Associations, Tachikawa Hospital, Tachikawa-shi, Tokyo, Japan; ^3^Department of Pathology, Federation of National Public Service Personnel Mutual Aid Associations, Tachikawa Hospital, Tachikawa-shi, Tokyo, Japan

## Abstract

We previously reported a case of giant cell carcinoma in the lung, in which the use of antiprogrammed death 1 (PD-1) immunotherapy resulted in substantial tumor reduction. In the present study, we describe an additional clinical course. A 69-year-old woman was diagnosed with giant cell carcinoma of the lung in clinical stage IVB (T2bN0M1c, BRA). The tumor expressed programmed death ligand 1 (PD-L1) in a high proportion. The patient received stereotactic radiotherapy for two sites of small brain metastases, followed by immunotherapy using anti-PD-1 antibodies (pembrolizumab). The treatment exerted a substantial tumor reduction through four cycles. However, treatment was withdrawn due to renal dysfunction. The primary lung tumor continued to regress for an additional four months without any further therapy, resulting in a clinical stage of T1aN0M0. Salvage thoracic surgery was then performed to remove the tumor residue in the lung. Microscopic examination of the sample revealed no residual cancer. The patient was free from recurrence at 16 months post surgery. We then comprehensively reviewed lung sarcomatoid carcinoma cases in the literature, in which anti-PD-1 antibodies were implemented. The current literature and our own findings suggest sarcomatoid carcinomas express high levels of tumoral PD-L1 and can be effectively treated with anti-PD-1 antibodies.

## 1. Introduction

The development of immune checkpoint inhibitors (ICIs) has helped improve the treatment of non-small-cell lung carcinomas (NSCLCs). However, immunotherapy utilizing ICIs only results in clinical benefits in a portion of treated patients and rarely results in complete clinical remission. The molecular and genetic background and histological type of the specific cancer can alter the disease immunogenicity and modify the therapeutic efficacy of ICIs. We previously reported on a case of giant cell carcinoma in the lung, which is a rare form of sarcomatoid carcinoma, in which a substantial tumor reduction was accomplished through the immunotherapy using pembrolizumab [1].

In this report, we describe the additional clinical course of the patient since we first reported on that case. The patient has shown a complete response to immunotherapy, which has been confirmed by surgical sampling. The patient has continued to experience an excellent clinical course and a long period of progression-free survival. We also comprehensively review the literature and discuss the potential benefits of ICI immunotherapy as a treatment course for sarcomatoid carcinomas.

## 2. Case Presentation

**Figure 1:**
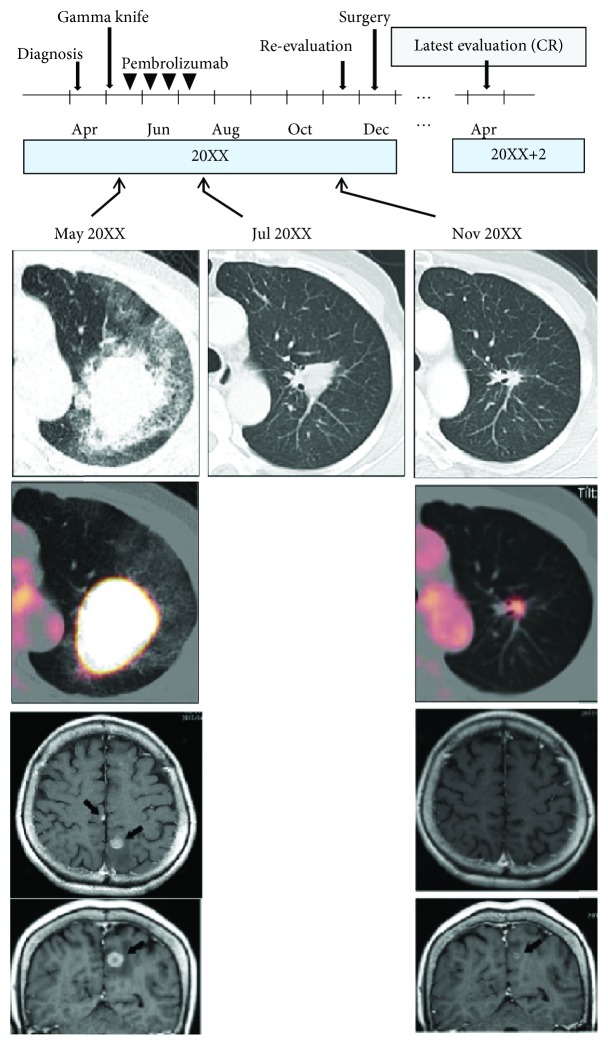
An outline of the clinical course is shown. CR: complete remission.

A 69-year-old Japanese woman was diagnosed with giant cell carcinoma in the lung at the clinical stage of IVB (cT2bN0M1c, BRA). Briefly, the primary tumor was located in the upper lobe of the left lung (37 mm in diameter), on which a 2-[^18^F]-fluoro-2-deoxy-D-glucose (FDG) positron emission tomography (PET) scan showed a high maximum standardized uptake value (SUV) of 28.4 (Figure 1). The PET scan also showed a marginal uptake of a maximum SUV of 3.49 in the mediastinum lymph nodes without any apparent enlargement. No other metastatic sites in the body were noted. A gadolinium-enhanced magnetic resonance imaging (MRI) scan detected two sites of small brain metastases (13 mm at the largest site) without any related neurological symptoms (Figure 1). A transbronchial biopsy aided in determining the pathological diagnosis of giant cell carcinoma. Stereotactic radiotherapy was indicated for the brain metastases in advance of implementing any anticancer medication. The primary tumor showed a high tumor proportion score (TPS) for programmed death ligand 1 (PD-L1) (75%). In response to this finding, the antiprogrammed death 1 (PD-1) antibody medication pembrolizumab (200 mg/body) was administered every three weeks for four cycles. Pembrolizumab exerted an obvious antitumor effect, and the primary tumor size decreased from 48 × 41 to 24 × 16 mm (a tumor reduction rate of 80.0%) at the end of the four cycles of treatment (Figure 1). However, a diagnosis of grade 2 renal dysfunction (Common Terminology Criteria for Adverse Events (CTCAE) v4.0) was noted and the treatment was discontinued after four cycles (see Reference [1] for more details).

Within 12 weeks of withdrawing pembrolizumab administration, renal function was restored to the pretreatment baseline without any corticosteroid use. From this point, the patient did not need any readministration of pembrolizumab as the primary lung tumor continued to regress on CT scans (7 × 7 mm in size), even after a four-month treatment-free period (Figure 1). The brain metastases were well-controlled after the stereotactic radiotherapy as assessed using MRI scans. The ring enhancement of the brain metastases on an MRI scan suggested radiation necrosis (Figure 1). A FDG-PET scan scheduled four months after discontinuing pembrolizumab revealed a moderate uptake of FDG on a portion of the residual primary tumor (maximum SUV of 4.01) (Figure 1). However, there was no significant uptake in the lymph nodes or in the extrathoracic organs. The reduction rate of maximum SUV in the primary tumor was calculated at 85.9%. These data together with radiological assessments revealed the lung cancer was now in yT1aN0M0, stage IA1.

**Figure 2:**
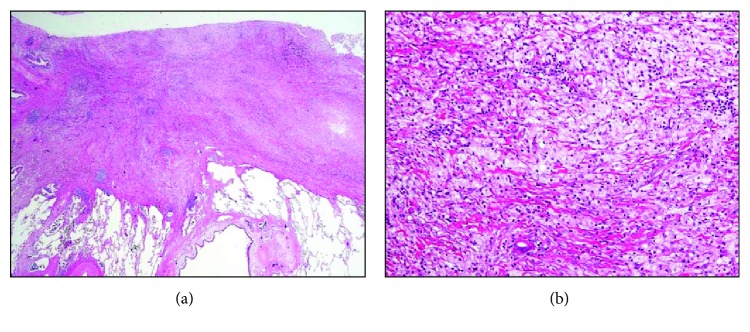
Pathological images taken with a microscope are shown in low (a) and high (b) magnifications. No residual cancer cells existed in the primary resected lung site. Instead, scar formation and an infiltration of mononuclear cells and foamy macrophages were observed.

The patient and her family were intensely interested in a salvage surgery to remove the cancer residue in the left upper lobe of the lung to avoid a tumor regrowth and to diminish the risk of future dissemination. In general, a salvage surgery for a patient in clinical stage IVB is not efficacious and not recommended as part of a standard therapeutic strategy. This is true even when a patient is downgraded to stage I after chemotherapy. However, in this particular case, there were some medical reasons for the patient to consider a salvage surgery, including (i) an FDG uptake on the primary lung tumor suggesting a cancer residue *in situ*, (ii) the rapid tumor progression during the initial evaluation period, (iii) the fact that giant cell carcinomas are generally resistant to chemotherapy with cytotoxic antitumor agents resulting in a poor prognosis, and (iv) the brain being the only metastatic site, where stereotactic radiotherapy can be highly effective. After a substantial discussion on the risks of salvage surgery and informed consent, which included a conversation addressing the potential for no clinical benefit, the patient decided to move forward with the procedure. The patient received a left upper lobectomy and a lymphadenectomy at the Federation of National Public Service Personnel Mutual Aid Associations, Tachikawa Hospital, in the following month after a full radiological evaluation (Figure 1). Pathological examination of the resected surgical specimens resulted in surprising findings, with no evidence that any cancer residue existed in the primary site under vigorous microscopic examination (Figure 2). Instead, nonspecific inflammation and scar formation were observed with an infiltration of lymphocytes, plasmacytes, and foamy macrophages (Figure 2). Lymph node metastasis was pathologically absent.

The patient experienced a loss of appetite and body weight loss beginning two months after the surgery and six months after a discontinuation of pembrolizumab. This was diagnosed as isolated adrenocorticotrophic hormone deficiency, grade 3 (CTCAE v4.0). It was presumed that the deficiency was attributable to an immune-related adverse effect of pembrolizumab and an increased demand for steroid hormones elicited by surgical stress. Corticosteroid replacement therapy conferred a rapid and total recovery from this condition. As of 16 months post surgery, the patient was free from any recurrence of cancer, as assessed using body CT and brain MRI scans, and was in good health.

## 3. Discussion

In the present study, we report on a rare case of giant cell carcinoma in the lung demonstrating a histologically complete response to anti-PD-1 immunotherapy. Additionally, multidisciplinary treatment including brain radiotherapy, immunotherapy, and a salvage thoracic surgery successfully induced a clinically complete remission. Due to the progressive nature of giant cell carcinomas in the lung, a median survival time in advanced-stage cases is reportedly 5-6 months when treated with cytotoxic antitumor agents [2, 3]. Given these findings, the present case represents an excellent clinical response and survival time as a result of the multidisciplinary treatments implemented in this study.

ICIs exert antitumor effects on cases with a wide variety of histological types in NSCLCs. Immunotherapy utilizing anti-PD-1/PD-L1 antibodies has been recommended as an option for the treatment of patients in advanced stages of cancer. However, it remains largely unknown whether lung carcinoma cases with minor histological types should be treated following the same strategy. It has become increasingly evident that lung sarcomatoid carcinomas present with a high tumoral PD-L1 expression (53-90% of all cases) [4–8]. This suggests a potential benefit of treating sarcomatoid carcinomas with PD-1/PD-L1 immunotherapy. We comprehensively searched the literature (written in English) for lung sarcomatoid carcinoma cases treated with anti-PD-1/PD-L1 inhibitors and summarized our findings in Table 1 [9–17]. Ten cases had pleomorphic carcinomas, and two cases had sarcomatoid carcinomas with unspecified subtypes. The present study was the only case of a giant cell carcinoma. PD-L1 expression was high (50-100% TPS), and anti-PD-1 antibodies (i.e., pembrolizumab or nivolumab) were used in all cases. The patients who were treated with nivolumab or pembrolizumab showed an objective response in 9 of 10 (90%) or in 3 of 3 (100%) cases, respectively. Two cases which received nivolumab treatment presented with a complete radiological response (Table 1). Further, treatment efficacy was not associated with any prior chemotherapy or thoracic surgery (recurrent cases). Given the findings of our literature review, we suggest the current case is unique in presenting a histologically complete response to anti-PD-1 immunotherapy in a case of giant cell carcinoma in the lung.

When investigating the efficacy of immunotherapy for the treatment of sarcomatoid carcinomas in other organs, we found the treatment of renal cell carcinomas (RCCs) particularly interesting. RCCs primarily manifest histologically as a type of clear cell carcinoma, while a sarcomatoid component only coexists in 5-8% of all cases [18]. It has been shown in histological examinations of resected tumors that PD-L1 expression is elevated in sarcomatoid RCCs when compared with nonsarcomatoid RCCs [19]. Interestingly, some cases have reported favorable clinical responses to nivolumab treatment in patients with sarcomatoid RCCs [20, 21]. However, to date, it has not been confirmed whether immunotherapy with ICIs is a superior therapy when compared to the standard treatment with cytotoxic antitumor agents in sarcomatoid RCCs.

In our review of basic research studies, we also noted that tumor mutation burden was recently proposed to be one of the predictors of ICI efficacy. For example, a study by Nakagomi et al. compared the genetic mutation burden between sarcomatoid and carcinomatous regions in four different lung sarcomatoid carcinoma cases. They showed that the sarcomatoid regions were more abundant in genetic mutations than in carcinomatous regions [22]. Given these findings and our current case report, we suggest ICIs targeting PD-1/PD-L1 could potentially play an important role in the antitumor treatment strategy of those patients with sarcomatoid carcinomas.

Another important observation we made was that a residual FDG uptake in a preoperative PET-CT scan was not associated with a pathological cancer residue in the present case. Recently, the immune PET Response Criteria in Solid Tumors (iPERCIST) was proposed to evaluate the metabolic response of solid tumors to immunotherapy [23]. In this assessment, a complete treatment response is defined as the disappearance of all metabolically active tumors. However, this approach could be difficult in evaluating cancer residues, since a restoration of antitumor immunity with ICIs can be associated with an upregulation of glucose metabolism. This could then lead to a misinterpretation on PET-CT scan images, as was shown in the present study.

In conclusion, we describe a giant cell carcinoma case in the lung which demonstrated a histologically complete response to PD-1/PD-L1 immunotherapy. A larger number of cases will be required to determine the potential role of immunotherapy in the treatment of sarcomatoid carcinomas in other patients and organs.

## Figures and Tables

**Table 1:** A summary of all case reports describing immunotherapy for lung sarcomatoid carcinomas.

	Age/sex	Pathology	PD-L1 TPS (%)	Tx line	Treatment	Response	Duration of ICI treatment	Ref
1	66/M	Pleomorphic	≧95	2nd	Nivolumab	PD	6 cycles/discontinue	[9]
2	59/F	Pleomorphic	80-90	3rd	Nivolumab	PR	19 cycles/ongoing	[9]
3	83/M	Pleomorphic	60-70	4th	Nivolumab	PR	10 cycles/ongoing	[9]
4	55/M	Pleomorphic	90	2nd	Nivolumab	PR	9 cycles/discontinue	[10]
5	62/M	Pleomorphic	70	2nd	Nivolumab	PR	12 cycles/ongoing	[11]
6	67/M	Pleomorphic	≧50	7th	Nivolumab	PR	8 cycles/ongoing	[12]
7	74/M	Pleomorphic	≧50	3rd	Nivolumab	PR	28 months/ongoing	[13]
8	75/M	Pleomorphic	90	3rd	Nivolumab	CR	3 cycles/ongoing	[14]
9	82/M	Pleomorphic	75	1st	Pembrolizumab	PR	3 cycles/discontinue	[15]
10	51/M	Pleomorphic	≧50	1st	Pembrolizumab	PR	3 cycles/ongoing	[16]
11	57/M	Sarcomatoid	80-90	1st (post operation recurrence)	Nivolumab	CR	3 months/ongoing	[17]
12	60/M	Sarcomatoid	100	1st (post operation recurrence)	Nivolumab	PR	8 months/ongoing	[17]
Present	69/F	Giant cell	75	1st	Pembrolizumab	CR	4 cycles/discontinue	—
